# Comparing Humoral and Cellular Adaptive Immunity during Convalescent Phase of COVID-19 in Hemodialysis Patients and Kidney Transplant Recipients

**DOI:** 10.3390/jcm10214833

**Published:** 2021-10-21

**Authors:** Dorota Kamińska, Hanna Augustyniak-Bartosik, Katarzyna Kościelska-Kasprzak, Marcelina Żabińska, Dorota Bartoszek, Paweł Poznański, Magdalena Kuriata-Kordek, Mariusz Kusztal, Oktawia Mazanowska, Magdalena Krajewska

**Affiliations:** Department of Nephrology and Transplantation Medicine, Wroclaw Medical University, 50-367 Wroclaw, Poland; dorota.kaminska@umed.wroc.pl (D.K.); hanna.augustyniak-bartosik@umw.edu.pl (H.A.-B.); marcelina.zabinska@umw.edu.pl (M.Ż.); dorota.bartoszek@umw.edu.pl (D.B.); pawel.poznanski@umw.edu.pl (P.P.); magdalena.kuriata-kordek@umw.edu.pl (M.K.-K.); mariusz.kusztal@umw.edu.pl (M.K.); oktawia.mazanowska@umw.edu.pl (O.M.); magdalena.krajewska@umw.edu.pl (M.K.)

**Keywords:** COVID-19, SARS-CoV-2, hemodialysis, kidney transplantation, humoral immunity, antibodies, cellular immunity, interferon-gamma release assay (IGRA)

## Abstract

Background. It is still unclear whether COVID-19 convalescent kidney transplant recipients (KTR) and hemodialysis (HD) patients can develop anti-SARS-CoV-2 adaptive immunity. The aim was to characterize and compare the immune response to the virus in HD patients and KTR. Methods. The study included 26 HD patients and 54 KTR—both convalescent (14 HD, 25 KTR) and unexposed. The immune response was assessed by determining the anti-SARS-CoV-2 antibodies in serum and specific T cell response via the interferon-gamma release assay (IGRA). Moreover, blood-morphology-derived parameters, immune cell phenotypes, and acute phase reactants were evaluated. Results. KRT and HD convalescents presented similar serum levels of anti-SARS-CoV-2 IgG and IgA. A negative correlation occurred between IgG and time after the infection was observed. There was a strong relationship between the prevalence of anti-SARS-CoV-2 cellular and humoral responses in both groups. Convalescent IGRA response was significantly higher in HD patients compared to KTR. Conclusions. HD patients and KTR develop humoral and cellular responses after COVID-19. The antibodies levels are similar in both groups of patients. SARS-CoV-2-reactive T cell response is stronger in HD patients compared to KTR. The SARS-CoV-2-specific IgG level decreases with time while IgA and a cellular response are maintained. IGRA proved to be a valuable test for the assessment of specific cellular immunity in immunocompromised HD patients and KTR.

## 1. Introduction

A pandemic outbreak of COVID-19, caused by a novel severe acute respiratory syndrome-related coronavirus named SARS-CoV-2 [1], has led to increased morbidity and mortality worldwide. Most of the patients do not present significant symptoms; however, approximately 15% develop a severe disease (with a need for oxygen support/hospitalization), while 5% develop a critical illness and require intensive care [2]. Some conditions and diseases predispose patients to severe COVID-19, e.g., advanced age, male gender, obesity, smoking, diabetes, hypertension, coronary heart disease, cerebrovascular disease, liver disease, chronic obstructive pulmonary disease, malignancies, and chronic kidney disease (CKD) [3].

Recent studies demonstrated that CKD is the strongest risk factor for severe COVID-19 with an odds ratio value of 2.31 [4] to even 2.97 [3]. Results from the ERA-EDTA Registry from 2020 indicated that COVID-19-attributed mortality was 20.0% for patients on dialysis and 19.9% for kidney transplant recipients (KTR) [5].

Whereas the majority of the general population (78–100%) develops an IgG antibody against SARS-CoV-2 within 10 to 21 days following infection, it is unclear whether HD patients and KTR with either natural or pharmacologically induced immunoincompetence will develop robust production of an antibody against SARS-CoV-2. McCafferty et al. showed a 95% seroconversion of hemodialysis patients following COVID-19 [6]. It was reported that despite a certain initial delay, transplant recipients can achieve serological and functional T cell immune responses comparable to the general population after COVID-19 [7]. However, Burack et al. reported that in solid organ transplant recipients with confirmed COVID-19, only 51% developed detectable anti-nucleocapsid IgG antibodies [8].

To fill the gap, we aimed to investigate the IgM, IgG, and IgA serological antibody responses as well as the SARS-CoV-2-reactive T cell responses in hemodialysis patients and kidney transplant recipients over the late convalescent clinical course after infection. We also included an in-depth analysis of the patients’ immune status to explain the reasons for the altered immune response to SARS-CoV-2 infection in these groups of patients.

## 2. Materials and Methods

### 2.1. Patients

Eighty adult patients (age > 18 years, 54.6 ± 12.4 years), including twenty-six individuals on maintenance hemodialysis (HD) and fifty-four kidney transplant recipients (KTR), from the Department of Nephrology and Transplantation Medicine of Wroclaw Medical University, were included in the study after their fully informed consent.

All the patients treated in the local hemodialysis unit were routinely tested every two to four weeks between April 2020 and November 2020 for the SARS-CoV-2 infection with automated nucleic acid amplification test on nasopharyngeal swab specimens (NPS), as part of the COVID-19 prevention program. The testing of HD patients was also performed in any case of suspicion of SARS-CoV-2 infection or exposure. KTR were tested when their SARS-CoV-2 infection was suspected.

The consecutive recruitment of KTR and HD patients was performed in March 2021 based on the following inclusion criteria:

- Age > 18 years,
- No clinical signs of COVID-19,
- If formerly tested positive for SARS-CoV-2, a minimum of 14 days must have passed since a positive test,
- Signed informed consent to participate in the study.

The exclusion criteria included:

- Previous anti-SARS-CoV-2 vaccination,
- Symptoms of any infection or neoplasm at the time of recruitment and during the preceding 2 weeks,
- In the case of kidney transplant recipients—histological signs of allograft rejection at the time of evaluation and in the preceding 4 weeks prior to the study.

Samples for immune testing were collected during in-person visits to our transplant or dialysis center.

The patients were classified as convalescent if:

- They had tested positive in any former rRT-PCR SARS-CoV-2 test regardless of presentation with COVID-19 symptoms
- And/or they were anti-SARS-CoV-2 seropositive at the time of recruitment.

The patients with no history of positive rRT-PCR SARS-CoV-2 test and without any detectable anti-SARS-CoV-2 antibodies were considered as probably unexposed and were included in the study as reference cases (12 HD, 29 KTR).

The enrollment was continued to reach the matching size of SARS-CoV-2 convalescent and reference subgroups among both KTR and HD.

The clinical characteristic of the recruited patients is presented in Table 1.

Twenty-three out of eighty patients included in the study had had a history of SARS-CoV-2 infection confirmed by rRT-PCR on nasopharyngeal swab specimens (11 HD, 12 KTR) before the study inclusion. Eight patients (4 HD, 4 KTR) had presented serious COVID-19 symptoms and required hospitalization, but none of them had suffered from severe respiratory failure with the need for mechanical ventilation. Of the 23 convalescent COVID-19 subjects tested, 21 (91%) were positive for antibodies against SARS-CoV-2 (IgG, IgA, or IgM antibodies) and 2 (7%) did not have detectable specific antibodies against SARS-CoV-2.

Sixteen patients (3 HD, 13 KTR) were classified as convalescent based solely on serum presence of anti-SARS-CoV-2 antibodies (serum sample classified as positive in at least one of the antibody subclasses), with no reported positive SARS-CoV-2 test before enrollment in the study.

The study was approved by the Bioethical Committee of Wroclaw Medical University (decision number KB–659/2020) and performed in accordance with the World Medical Association Declaration of Helsinki.

### 2.2. Routine Diagnostic Methods

The general inflammation-related markers from peripheral blood were assessed (C-reactive protein (CRP), hemoglobin, albumin, brain natriuretic peptide (BNP), N-terminal pro-b-type natriuretic peptide (NT-pro-BNP)). Blood morphology-derived parameters obtained from 5-part differential blood count, as simple indicators of the inflammatory response to various stimuli—NLR (neutrophil–to–lymphocyte ratio), dNLR (derived neutrophil–to–lymphocyte ratio), PLR (platelet–to–lymphocyte ratio), and MLR (monocyte–to–lymphocyte ratio)—were also included in the study.

### 2.3. SARS-CoV-2 rRT-PCR

The patients were tested for SARS-CoV-2 infection by a government-accredited COVID-19 laboratory. The infection was confirmed with SARS-CoV-2 automated nucleic acid amplification testing of NPS with a real-time reverse transcription-polymerase chain reaction, rRT-PCR, for ORF 1ab and N genes. In most cases, a test was performed in a local diagnostic laboratory with a PCR SARS-CoV-2 Real-Time PCR LAB-KIT (Biomaxima, Lublin, Poland) with the QuantStudio 6 Flex (Applied Biosystems, Waltham, MA, USA).

### 2.4. SARS-CoV-2 Specific Antibodies

The humoral immune response was assessed by determining the presence of anti-SARS-CoV-2 IgG, IgA, and IgM antibodies in serum samples. Concentrations of serum anti-SARS-CoV-2 IgG were measured using quantitative enzyme-linked immunosorbent assay (anti-SARS-CoV-2 QuantiVac ELISA, EUROIMMUN) according to manufacturer protocol. The detection of IgG antibodies against SARS-CoV-2 is based on the S1 domain of the spike protein, including the immunologically relevant receptor-binding domain (RBD) as an antigen. RBD represents important target antigens for virus-neutralizing antibodies.

The test results of the measurement were converted into standardized binding anti-body units (BAU/mL), which comply with the First WHO International Standard for anti-SARS-CoV-2 immunoglobulin (NIBSC 20/136). The manufacturer recommends the following interpretation of the results for IgG in serum: <25.6 BAU/mL negative, ≥25.6 BAU/mL and <35.2 BAU/mL borderline, and ≥35.2 BAU/mL positive.

The presence of serum anti-SARS-CoV-2 IgA and IgM was measured using semi-quantitative ELISA (EUROIMMUN) according to manufacturer protocols. According to the manufacturer’s guidelines, the results are presented as a ratio to the cutoff value, with the results for the serum samples to be interpreted as follows: <0.8 negative, ≥0.8 and <1.1 borderline, and ≥1.1 positive. The S1 domain of the spike protein is the antigen used in the ELISA for the detection of IgA. The ELISA for the detection of IgM antibodies against SARS-CoV-2 is based on the modified nucleocapsid protein (NCP).

### 2.5. SARS-CoV-2 Cellular Response

SARS-CoV-2-reactive T cell responses were evaluated using a SARS-CoV-2 interferon-gamma release assay (IGRA, EUROIMMUN) according to the manufacturer’s protocol. The assay analyzes interferon-gamma production by T cells after peripheral blood sample stimulation with SARS-CoV-2 S1 domain of spike glycoprotein. The test results are presented as a level of interferon-gamma detected after antigen stimulation, corrected for its basal plasma level.

### 2.6. Immune Cell Phenotypes

The following mouse anti-human antibodies (Becton Dickinson) were used for cell phenotyping: anti-CD3-APC (UCHT1), anti-CD4-PerCP (SK3), anti-CD25-FITC (M-A251), anti-FOXP3-PE (259D/C7), anti-CD8-APC-Cy7 (SK1), anti-CD28-PE (L293), anti-CD57-FITC (HNK-1), anti-CD19-PerCP-Cy5.5 (HIB19), anti-CD20-APC-H7 (L27), anti-CD27-PE-Cy7 (M-T271), anti-CD38-APC (HIT2), anti-CD24-PE (ML5), anti-IgD-FITC (IA6-2), Human Th1/Th2/Th17 Phenotyping Kit, and the four-color BD Multitest (CD3/CD16+CD56/CD45/CD19).

The absolute number of T cells per µL of blood was determined with BD Multitest in Trucount tubes (Becton Dickinson) according to manufacturer protocol. The below-described subpopulations were measured in relation to T or B cells, which also enabled their enumeration per µL of blood.

The samples of heparin-anticoagulated blood were stained in 4 panels:

- Regulatory T cells—anti-CD25-FITC, anti-FOXP3-PE, anti-CD4-PerCP, and anti-CD3-APC; whole blood staining, lysed with BD FACS Lysing Solution, followed by Human FoxP3 Buffer Set fixation and permeabilization;
- Th and Tc lymphocytes—anti-CD57-FITC, anti-CD28-PE, anti-CD4-PerCP, anti-CD3-APC, anti-CD8-APC-Cy7; whole blood staining, lysed with BD FACS Lysing Solution and washed with BD Pharmingen Stain Buffer (FBS);
- Th1, Th2, and Th17—blood samples stimulated for 5 h with PMA and ionomycin in the presence of GolgiStop BD, followed by staining with Human Th1/Th2/Th17 Phenotyping Kit;
- B cells—anti-IgD-FITC, anti-CD24-PE, anti-CD27-PE-Cy7, anti-CD19-PerCP-Cy5.5, anti-CD38-APC, and anti-CD20-APC-H7); whole blood staining, lysed with BD FACS Lysing Solution and washed with BD Pharmingen Stain Buffer (FBS).

All samples were analyzed using a six-color FACSLyric flow cytometer and FACSSuite software (Becton Dickinson).

### 2.7. Statistical Analysis

Descriptive statistics, including mean ± SD, median, and interquartile range, were calculated for all demographics, clinical characteristics, and laboratory data. The normality of data distribution was verified with the Shapiro–Wilk normality test. Since most of the data did not fit a normal distribution, non-parametric statistics were employed. Intergroup comparisons of continuous data were assessed using the non-parametric Mann–Whitney U test. The correlations were performed using rank correlation (Spearman). The frequencies were compared using the exact Fisher test. A receiver operating characteristic (ROC) curve was used to define the cutoff value for IGRA response related to SARS-CoV-2 convalescence. The statistical test results, for which the *p*-values were less than 0.05, were considered significant. Statistical analysis was performed using Statistica (data analysis software system), version 13 (TIBCO Software Inc. Palo Alto, CA, USA) and MedCalc (version 20, MedCalc Software Ltd., Ostend, Belgium).

## 3. Results

### 3.1. Anti-SARS-CoV-2 Antibodies in Convalescent Hemodialysis and Transplant Patients

According to the applied classification rule, the reference HD and KTR patients had not presented a detectable level of any subclass of anti-SARS-CoV-2 antibodies at the time of sampling. 

The observed levels of anti-SARS-CoV-2 antibodies in kidney transplant convalescent patients are presented in Table 2. Most of them had SARS-CoV-2-specific IgG and IgA antibodies and only 3 had IgM as well. In the case of KTR who were anti-SARS-CoV-2 seropositive with no history of positive rRT-PCR test result nor recorded symptoms of COVID-19, the observed level of IgG antibodies was lower than in the case of recipients with confirmed former SARS-CoV-2 infection (*p* = 0.016).

The observed levels of anti-SARS-CoV-2 antibodies in hemodialysis convalescent patients are presented in Table 3. Most of them had SARS-CoV-2-specific IgG and IgA antibodies and only 2 had IgM as well. There was a low number of hemodialysis patients whose infection was not confirmed with rRT-PCR; however, the observed antibody concentrations for them were similar to those for the confirmed subgroup of the patients.

Generally, we found no difference between specific anti-SARS-CoV-2 IgG levels in the convalescent HD patients compared to KTR (median, IQR: HD—534, 106–834, KTR—207, 106–510 BAU/mL, *p* = 0.626). Additionally, the anti-SARS-CoV-2 IgA level observed for the convalescent patients did not differ between the groups (median, IQR: HD—6.2, 3.7–19.9, KTR—8.1, 4.2–29.6, *p* = 0.580). We analyzed the association between the anti-SARS-CoV-2 antibodies and time since the first positive rRT-PCR SARS-CoV-2 test. The respective serum samples were collected between 19 and 267 days after a positive rRT-PCR test (74, 47–106 days). We observed a negative correlation between IgG antibodies and time since the first positive rRT-PCR test (rs = −0.45, *p* = 0.039), while the IgA antibody level was not shown to be time-related over the analyzed period (rs = −0.30, *p* = 0.188).

### 3.2. Anti-SARS-CoV-2 Cellular Response

**Figure 1:**
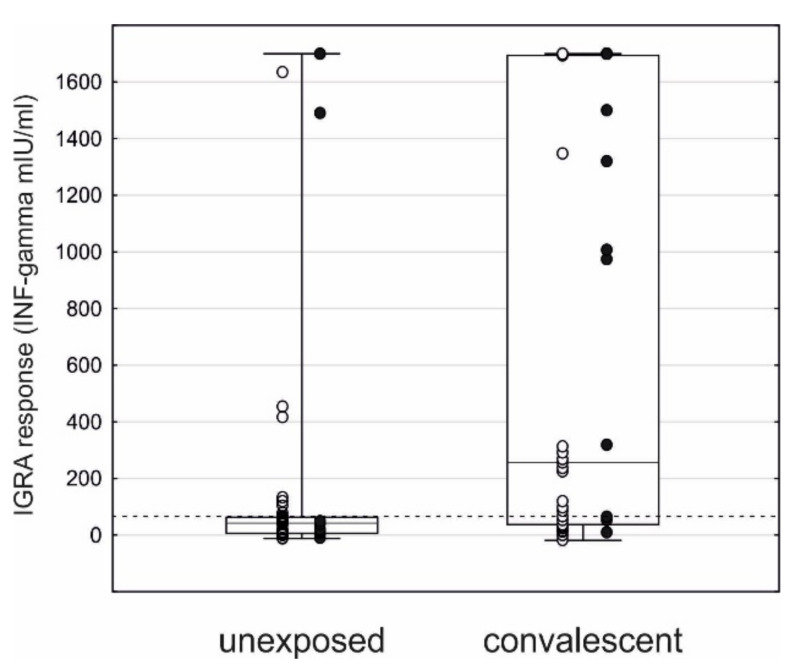
IGRA response in SARS-CoV-2 convalescent (median, IQR: 257, 37–1693 mIU/mL) and reference patients (42, 6–64, *p* < 0.001). Raw data points: ○ KTR, ● HD.

The observed level of cellular response detected with IGRA was higher in the convalescent patients compared to the reference ones (median, IQR: 257, 37–1693 vs. 42, 6–64, *p* < 0.001, Figure 1), both HD (1410, 318–1700 vs. 17, 6–44, *p* = 0.001), and KTR (97, 29–291 vs. 49, 8–65, *p* = 0.040). 

We did not observe any difference in IGRA results between convalescent rRT-PCR-confirmed HD cases and unconfirmed ones (Table 4).

We performed the ROC analysis to estimate the IGRA cutoff value related to SARS-CoV-2 exposure in the HD group. We used convalescent HD individuals as cases and reference HD patients as controls. The ROC analysis provided a cutoff value of 64 mIU/mL for defining a positive T cell response against SARS-CoV-2 (AUC 0.86, *p* < 0.001, sensitivity 79%, specificity 83%).

We have shown in Section 3.1 that there was no difference in anti-SARS-CoV-2 antibody levels between hemodialysis and transplant patients. On the contrary, we observed that SARS-CoV-2-reactive T cell response measured by IGRA in the convalescent group was significantly higher in HD patients compared to KTR (1410, 318–1700 vs. 97, 29–291 mIU/mL, *p* = 0.009). As in the case of HD patients, convalescent rRT-PCR-confirmed KTR did not show significantly different IGRA results compared to unconfirmed ones (Table 4).

The ROC analysis was also statistically significant in the case of KTR (AUC 0.66, *p* = 0.032), and the cutoff value of 64 mIU/mL presented a sensitivity of 60% and a specificity of 72% for detecting previous SARS-CoV-2 exposure via the assessment of T cell responses.

When 64 mIU/mL cutoff was applied, the SARS-CoV-2-specific T cellular response was observed in 78.6% of rRT-PCR and/or seropositive HD cases and in 60.0% of respective KTR (*p* = 0.206).

Our data did not show a relationship between the IGRA response and time since the first positive rRT-PCR test (HD—rs = −0.246, *p* = 0.473; KTR—rs = −0.30, *p* = 0.393).

### 3.3. SARS-CoV-2 Specific Immune Characterization of the Patients

We found a strong relationship between the prevalence of anti-SARS-CoV-2 cellular and humoral responses in both HD and KTR groups of patients.

IGRA response with a level above the cutoff value of 64 mIU/mL was observed in 11 out of 13 seropositive HD patients and in only 2 out of 13 negative ones (*p* < 0.001). However, there was no relationship between the quantitative measures of both types of responses in HD convalescent patients (IGRA vs. IgG level: rs = 0.16, *p* = 0.584; IGRA vs. IgA level: rs = 0.39, *p* = 0.169).

Eight rRT-PCR-confirmed convalescent HD patients presented detectable levels of both anti-SARS-CoV-2 humoral and cellular responses; in two cases, only specific antibodies were detected, and in one patient no response was observed. All three HD patients included in the study as convalescents based on their specific humoral response also proved positive in IGRA. In our convalescent HD group of patients, 78.6% of patients mounted a detectable level of both types of specific anti-SARS-CoV-2 response, while 14.3% only mounted a humoral one.

The SARS-CoV-2-specific cellular response was also observed in 15 out of 24 seropositive KTR and in 8 out of 30 negative ones (*p* = 0.009). There was a correlation between the IGRA response measure and IgG level in this group of patients (rs = 0.49, *p* = 0.013). As in the case of HD, IgA level was unrelated to IGRA response in KTR (rs = 0.30, *p* = 0.148).

Nine rRT-PCR-confirmed convalescent KTR presented detectable levels of both anti-SARS-CoV-2 humoral and cellular responses; in two cases, only specific antibodies were detected, and in one patient no response was observed. From KTR included in the study as convalescents based on their specific humoral response, six also proved positive in IGRA, while seven did not respond in a cellular assay. In our group of convalescent KTR, 60.0% of patients mounted a detectable level of both types of specific anti-SARS-CoV-2 response, while 36.0% mounted only a humoral one. Although there were slightly more KTR responding only in a humoral manner than HD patients, this observation was not statistically significant.

We observed a detectable level of IGRA response in 8 KRT and 2 HD patients considered unexposed due to a lack of specific anti-SARS-CoV-2 antibodies or records of positive nucleic acid amplification or antigen tests. The two HD patients presented a high concentration of released interferon-gamma (1491 and 1700 mIU/mL), while KTR was in the range of 65–1636 mIU/mL (median, IQR: 126, 91–434 mIU/mL).

**Figure 2:**
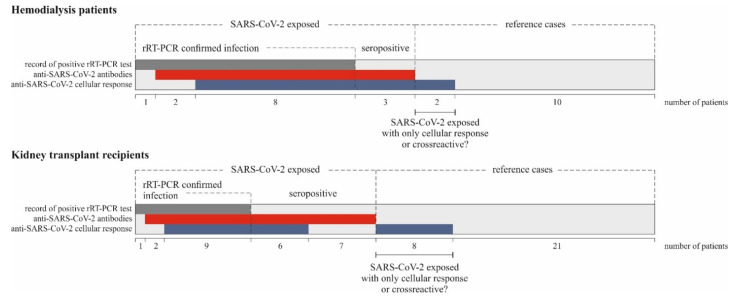
Observed type of SARS-CoV-2 specific response in KTR and HD patients included in the study.

The overall SARS-CoV-2 specific immune response observed in KTR and HD patients included in the study is summarized in Figure 2.

### 3.4. Analysis of Standard Laboratory Parameters and Inflammatory Markers in Relation to Convalescent SARS-CoV-2 Infection

Hemodialysis and transplant population differed significantly in terms of standard laboratory parameters (Supplementary Tables S1–S2), so we analyzed the influence of SARS-CoV-2 infection on their levels in each patient group. No influence of convalescent SARS-CoV-2 infection was found in the renal transplant group. Convalescent hemodialysis patients presented significantly higher CRP (median, IQR: 6.5, 3.6–17.7 vs. 3.3, 1.9–4.6 mg/L, *p* = 0.041) and lower albumin levels (3.4, 3.1–3.9 vs. 4.0, 3.7–4.2 g/dL, *p* = 0.013) than the unexposed HD group.

Additionally, the HD and KTR groups differed significantly in the values of inflammatory markers derived from blood morphology parameters (NLR—3.88, 2.22–5.89 vs. 1.72, 1.33–2.23, *p* < 0.001; PLR—128, 102–186 vs. 88, 73–104, *p* < 0.001; and MLR—0.51, 0.38–0.61 vs. 0.31, 0.25–0.39, *p* < 0.001; Supplementary Tables S1–S2). As in the case of standard laboratory parameters, no impact of convalescent SARS-CoV-2 infection on inflammatory markers was observed in KTR. However, convalescent HD patients presented significantly higher PLR (156, 128–208 vs. 104, 89–126, *p* = 0.010) and MLR (0.50, 0.49–0.63 vs. 0.39, 0.28–0.52, *p* = 0.012) compared to unexposed individuals.

### 3.5. Analysis of Lymphocyte Subpopulations

HD patients presented lower general lymphocyte count than KTR (1.4, 1.0–1.5 vs. 2.7, 2.0–3.1, *p* < 0.001), which was further reflected in a lower count of T, CD4+ T, CD8+ T, B, NK, and NKT cells (Supplementary Tables S3–S6). A more detailed lymphocyte phenotyping revealed that despite a higher count of T cells in KTR, the size of regulatory T cells, Th1, Th2, and Th17 subpopulations was not increased in this group of patients compared to HD. Moreover, KTR were characterized by low counts of transitional B cells and plasmablasts compared to HD. Since a distribution of the lymphocyte subpopulations differed between the groups of patients, the analysis of COVID-19 exposure impact was performed separately for KTR and HD patients. We found no differences between convalescent and unexposed transplant recipients. Hemodialysis convalescent patients revealed significantly decreased double-positive CD4+CD8+ T cell count compared to the negative group (7.4, 6.5–9.6 vs. 19.2, 9.9–35.8 cells/µL, *p* = 0.001).

## 4. Discussion

COVID-19 is a serious and potentially lethal infection for patients with chronic kidney disease, especially hemodialysis patients or kidney transplant recipients. It is unclear if CKD patients can mount an effective anti-SARS-CoV-2 directed adaptive immune response.

Patients treated with dialysis or kidney transplantation represent a population with high risk and poor outcomes of COVID-19. Patients with chronic kidney disease are exposed to SARS-CoV-2 infection due to frequent contact with health services at outpatient clinics, dialysis centers, and hospitals.

Even without a pandemic risk, the mortality rates in dialysis patients are more than eight times higher than in the general population. Kidney transplant recipients experience 30–50% reduced life expectancy [5]. Infections are the second main cause of death in patients with kidney failure, with a mortality rate of between 12% and 22% [9].

Although the incidence among kidney transplant recipients seems to not differ from the general population, the COVID-19-related mortality reported in this group of patients ranges from 17.9% to 28% [10,11]. This particularly applies to elderly kidney transplant recipients, presenting a 50% short-term fatality rate [12]. A recent large study showed that the risk of COVID-19-related death was 78% higher in kidney transplant recipients compared with hemodialysis patients, especially during the first post-transplant year [13].

The ability of hemodialysis patients and transplant recipients to mount a protective antiviral response, despite the immunosuppression state, would be crucial during SARS-CoV-2 infection.

We analyzed the SARS-CoV-2 specific immune response in a group of 25 KTR and 14 HD convalescent patients. 91.3% of the study patients with rRT-PCR-confirmed SARS-CoV-2 infection developed anti-SARS-CoV-2 antibodies specific to the S1 spike protein. We found no difference between specific anti-SARS-CoV-2 IgG or IgA levels in the convalescent HD or KTR groups of patients. The IgG antibody titer was negatively correlated with time since the first positive rRT-PCR test, supporting the hypothesis of its decline with time post-infection. The IgA antibody level was not shown to be time-related over the analyzed period.

In the study published by La Milia et al., all COVID-19-symptomatic survivors maintaining dialysis developed anti-SARS-CoV-2 spike-specific IgG, with a peak at the third month and then a slow reduction until the sixth month. Among asymptomatic survivors, only 7% were seropositive 2 months after the positive rRT-PCR [14]. Further small studies also reported a 100% seroconversion rate in HD patients after COVID-19 [15,16,17]. Forbes et al. published that 95% of hemodialysis patients mount an antibody response following COVID-19 [18]. No significant decline of antibody level over 145 days of observation was reported in that hemodialysis population. Alfano et al. recently presented a case report of three hemodialysis patients maintaining anti-SARS-CoV-2 neutralizing antibodies for a year since COVID-19 [19]. Those reports are in line with our observation that 90.9% of PCR-confirmed convalescents in the HD group were able to mount a humoral response to SARS-CoV-2 infection.

In German kidney transplant recipients, the seroprevalence of SARS-CoV-2 antibodies during systematic antibody screening was low, but all COVID-19 patients underwent a seroconversion 4 to 7 weeks after onset of the disease [20]. An in-depth analysis revealed that 88% of kidney transplant recipients who survived COVID-19 were positive for anti–spike IgG antibodies, and only 28% were positive for anti-nucleocapsid antibodies [21]. However, another study showed that the vast majority of KTR can mount anti-SARS-CoV-2 anti-nucleocapsid IgG in the convalescent phase [22]. Benotmane et al. reported that, despite an extended time of viral shedding in KTR, the antibody kinetics are similar to those observed in immunocompetent subjects, with a peak at day 40 [23]. 72.4% were found to display anti-SARS-CoV-2 antibodies up to 6 months after COVID-19. One small study on 10 organ transplant recipients revealed that anti-SARS-CoV-2 spike-specific neutralizing antibodies did not differ from healthy controls. A decline and loss of anti-SARS-CoV-2 anti-nucleocapsid antibodies in kidney transplant recipients were reported after the 6 months following SARS-CoV-2 infection [24]. Another study on 21 KTR showed that the majority of them exhibited detectable SARS-CoV-2-specific cell-mediated immunity 6 months after infection, with an 89.5% seroconversion rate [25]. In our study, the vast majority of rRT-PCR-confirmed convalescents KTR (91.7%) mounted a humoral response to SARS-CoV-2 infection, which confirms the abovementioned reports.

The available data from reports on post-COVID-19 immunity, also supported by those regarding anti-SARS-CoV-2 vaccination, indicate that short time after transplantation, which is an equivalent of a higher immunosuppression burden, is the most important factor responsible for reduced mounting of the anti-SARS-CoV-2 immune response within the first post-transplant months [26]. Our KTR were median 47, IQR 20–102 months after transplantation, so the immunosuppression-related drawback in a specific immune response may not have been pronounced.

Moreover, the sensitivity of different immune assays used for the assessment of anti-SARS-CoV-2 humoral response may be responsible for some discrepancies of reported seropositivity rates in particular groups of patients.

Our study also included the assessment of SARS-CoV-2 directed T cell responses in kidney transplant recipients and HD patients using the interferon-gamma release assay (IGRA, EUROIMMUN). The IGRA used employs whole blood samples for the determination of T cell activity against SARS-CoV-2 via sample activation with the S1 spike antigen followed by detection of interferon-gamma released by stimulated antigen-specific T cells.

We performed the ROC analysis with convalescent individuals as cases and reference ones as controls to estimate the IGRA cutoff value related to SARS-CoV-2 exposure. The ROC analysis provided a cutoff value of >64 mIU/mL for defining a positive T cell response against SARS-CoV-2. When this cutoff was applied, 79% of HD convalescent patients and 60% of convalescent KTR presented a detectable cellular response. SARS-CoV-2-reactive T cell response measured by IGRA was significantly higher in hemodialysis patients compared to the transplant population.

Interferon-gamma release assay based on EUROIMMUN products was reported to successfully detect broad T cell reactivity against the structural SARS-CoV-2 proteins in the general population [27]. The mentioned study presented an analysis of T cell responses for specific stimulation with four different SARS-CoV-2 antigenic regions, including spike protein fragments. We used the only commercially available stimulation set, which included the S1 domain of the spike protein as a specific stimulus.

The authors of the above study proposed a single cutoff value of >40 mIU/mL for all antigenic fragments in the general population. We were able to confirm that a slightly higher cutoff of >64 mIU/mL is optimal for the assessment of the S1 domain antigenic stimulation in CKD patients.

Although some data regarding T cell-mediated immunity in the general population is available, only a few studies investigated cellular anti-SARS-CoV-2 response in CKD patients using flow cytometry or laborious ELISPOT assays. T cell immunity in CKD patients is disturbed with a decrease in newly formed T cells and changes in T cell differentiation leading to accelerated immune aging [28]. It has also been reported that kidney transplantation does not reverse this process [29]. In immunocompetent patients, SARS-CoV-2-specific T cell immunity is sustained for at least 6 months following primary infection [30]. T cell response against SARS-CoV-2 in immunocompromised individuals, including HD patients and kidney transplant recipients, is still a subject of debate.

In a study on hemodialysis patients, a positive anti-SARS-CoV-2 cellular response was found in 8 out of 11 patients six months after COVID-19 with the use of the T-SPOT discovery SARS-CoV-2 (Oxford Immunotec) assay [31]. Thieme et al. reported that frequencies of anti-nucleocapsid-protein-reactive and anti-SARS-CoV-2 polyfunctional CD8 T cells assessed by flow cytometry did not differ in 10 organ transplant recipients from non-immunocompromised convalescents [32]. A study on 21 KTR showed that approximately 57% of them presented detectable SARS-CoV-2-specific cell-mediated immunity 6 months after infection (more often CD4+ T cell than CD8+ T cell response), as determined by flow cytometry and IFN-γ FluoroSpot (Mabtech) assay [25]. A study including 28 solid organ transplant recipients revealed that unlike during acute infection, there were no differences between transplant recipients and non-immunocompromised convalescents regarding the distinct SARS-CoV-2-reactive T cell response detected by the FluoroSpot Immune assay kit (AID Gmbh) [7]. Additionally, one small study on SARS-CoV-2-specific T cell-response revealed no difference between KTR (*n* = 5) and hemodialysis patients (*n* = 2) approximately 1 month after the infection with the ELISPOT assay [33]. SARS-CoV-2-reactive CD8+ T cells targeting membrane and spike proteins tended to be lower in transplant recipients, in contrast to anti-nucleocapsid-protein-reactive and anti-SARS-CoV-2 polyfunctional CD8+ T cells [32].

In our study, 78.6% of convalescent HD patients and 60.0% of convalescent KTR presented detectable levels of both types of specific anti-SARS-CoV-2 response. 14.3% of convalescent HD patients and 36.0% of KTR proved only seropositive without a detectable level of IGRA response, but this observation was not statistically significant. The above is in line with published reports [7,25,31] showing that a vast majority of HD and more than half KTR can mount either a humoral or cellular response to SARS-CoV-2 infection.

Most studies on vaccination-induced anti-SARS-CoV-2 immunity showed an impaired response among kidney transplant recipients, whereas HD patients reached almost healthy-control levels [34,35,36,37]. A recent study showed that after two doses of mRNA vaccination, >95% of dialysis patients presented anti-S IgG antibodies, while only 63.3% of KTR seroconverted with substantially lower antibody levels. An anti-SARS-CoV-2 cellular response was observed for 77.6% hemodialysis patients and 61.3% of KTR transplanted more than one year ago, while only for 36% of those transplanted within the previous 12 months [26].

In the case of our KTR group (median 47, IQR 20–102 months after transplantation), the immunosuppression-related drawback in the generation of a specific cellular response may not be pronounced. However, although they were able to induce specific anti-SARS-CoV-2 reactive cells upon infection, the level of S1 antigen-induced interferon-gamma release during the convalescent phase was significantly lower than that observed for HD patients.

It should be noted that we observed a detectable level of IGRA response in eight KRTs and two HD patients considered probably unexposed due to a lack of specific anti-SARS-CoV-2 antibodies or records of positive nucleic acid amplification or antigen tests. The patients presented a concentration of released interferon-gamma in the range of 65–1700 mIU/mL (median, IQR: 274, 103–1490 mIU/mL). We did not find for those patients any common clinical characteristics, such as underlying kidney disease, immunosuppression treatment, autoimmune disorders, viral hepatitis, duration of hemodialysis, or time after transplantation.

Although none of our rRT-PCR-confirmed cases presented IGRA response without concurrent antibody presence, it can be assumed that those patients could have also experienced SARS-CoV-2 infection and responded mostly in a cellular way or that antibody level dropped out below the detection limit over time.

On the other hand, EUROIMMUN has not provided the cross-reactivity analysis for the offered IGRA, so it cannot be ruled out that despite stimulation based on the highly SARS-CoV-2-specific S1 domain, a cross-reactivity with, e.g., common cold-causing coronaviruses occurred in some of the cases.

Grifoni et al. analyzed the T cell-mediated responses towards pools of peptide fragments of SARS-CoV-2 proteins [38]. They observed some reactivity in unexposed subjects, which suggested the presence of cross-reactive coronavirus-specific T cells for at least some antigenic fragments.

It was also reported in another study based on the in-house developed IGRA that a significant proportion of healthy unexposed individuals had a specific and strong T cell response determined with the production of interferon-gamma after stimulation with SARS-CoV-2 specific antigen [39].

We also performed a broad analysis of peripheral blood lymphocytes, standard laboratory parameters, and immune markers to reveal the post-COVID-19 patterns in KTR and HD patients.

Generally, hemodialysis patients are characterized by uremia-related immunological abnormalities, such as decreased general blood counts and malfunction of immune cells [40]. Blood morphology-derived parameters obtained from five-part differential blood count were shown to be altered in various inflammatory states, including COVID-19 [41], which was also observed for CKD COVID-19 patients [42,43,44]. Renal dysfunction is associated with an increase in endothelial injury-related BNP and NT-pro-BNP markers that were found to be associated with disease severity and mortality in COVID-19 patients [45]. Kidney transplantation reverses some, but not all, of the deficiencies of the immune response within the first post-transplant year.

In our group of convalescent HD patients, significantly higher PLR and MLR could be observed compared to unexposed patients. We also noted significantly higher CRP with lower albumin levels in convalescent HD patients compared to non-infected individuals, which may all reflect the previous activation of immune response in SARS-CoV-2-infected patients.

COVID-19 is characterized by heterogeneity of the immune response, and the observed immunotypes may reflect fundamental differences in how patients respond to SARS-CoV-2 infection [46]. Although COVID-19 may result in very deep activation and/or exhaustion of immune cell populations, most of the observed variations are temporary and are reversed in convalescent individuals [46,47].

In the current research, we observed the general immunophenotyping results similar to those obtained in our pre-COVID-19 study, both for HD patients and KTR [48]. We did not observe any influence of the preceding exposure to SARS-CoV-2 on immune cell phenotypes in KTR. However, in convalescent HD patients, we noted a decreased count of double-positive CD4+CD8+ T cells, a poorly studied population with varying functions suggested in different clinical conditions [49], which may be considered the only visible reminiscence of the virus-related immune variations in this group of patients. Our results suggest that despite the immunocompromised status, KTR and HD patients can return to their basal immune state following SARS-CoV-2 infection.

Overall, we showed that chronic kidney disease patients on maintenance hemodialysis or after kidney transplantation can mount humoral and T cell-mediated response after COVID-19 infection. We also observed that T cell-mediated response might be preserved longer than humoral response.

Moreover, a maintenance immunosuppressive treatment after kidney transplantation seems not to hamper significantly the production of specific anti-SARS-CoV-2 antibodies or generation of specific T-cells. However, it influences the S1 antigen responsiveness of the specific T cells, which is lower compared to hemodialysis patients.

### Limitation of the Study

Our study was performed on a group of 54 KTR and 26 HD patients from one transplant and dialysis center; however, the study size group exceeded those in already published studies. We focused not only on confirmed COVID-19 patients but analyzed a group of our KTR and HD patients to follow previously undetected asymptomatic SARS-CoV-2 exposures as well. As a result, we do not have precise data regarding the time of SARS-CoV-2 exposure for some of the patients.

As there is a significant amount of data regarding general population response to SARS-CoV-2 infection, we focused only on the patients and did not include healthy controls for comparison. The enrolled patients were anti-SARS-CoV-2-vaccinated in a broad vaccination program for hemodialysis patients and transplant recipients in a short time following the enrollment in the study, which did not allow us to follow up the solely post-infection immune response over the time in these groups of patients.

As the EUROIMMUN IGRA is for research use only and no validation data are available in the manufacturer release, we cannot exclude any potential cross-reactivity of T cell responses.

We did not analyze the SARS-CoV-2 variants’ specific responses. The patients were sampled in March 2021, and it can be expected that they had been mostly exposed to initial variants of the virus (before the spread of B.1.1.7 in Poland).

## 5. Conclusions

Our study provided data on SARS-CoV-2-reactive humoral and cellular adaptive immunity in chronic kidney disease patients on maintenance hemodialysis or kidney transplant recipients in the convalescent phase after COVID-19. The main findings of our study are as follows:

- Hemodialysis patients and kidney transplant recipients can induce both a humoral and cellular response after SARS-CoV-2 infection. The specific anti-SARS-CoV-2 IgG level is not different between the groups of patients, as opposed to SARS-CoV-2-reactive T cell response (IGRA), which is stronger in the hemodialysis patients compared to the transplant population.
- The SARS-CoV-2-specific IgG antibody level decreases with time, while IgA and the specific anti-SARS-CoV-2 cellular response is maintained for a longer time.
- The interferon-gamma release assay is a valuable test for the assessment of T cell-mediated immunity in immunocompromised KTR and HD patients.

The knowledge regarding SARS-CoV-2 post-infection response in HD patients and kidney transplant recipients is still not very well-established. Our study adds new data in this field, particularly to the very scarce published data on post-infection cellular response in these patient groups. Moreover, due to prolonged viral shedding [50], COVID-19 in immunocompromised patients can increase the risk of the development of SARS-CoV-2 escape variants that can spread in the general population. In this context, studies on the immune response to SARS-CoV-2 in immunocompromised patients are important not only for this population but also for the general community.

## Figures and Tables

**Table 1:** Clinical characteristics of the recruited patients.

	KTR	HD
Number of patients	54	26
Gender (F/M)	27/27	17/9
Age (years)	52.9 ± 11.4; 54, 45–62	58.4 ± 13.7; 61, 46–70
BMI (kg/m^2^)	25.6 ± 4.1; 26, 24–28	26.8 ± 5.6; 26, 22–31
Blood type		
0	17	12
A	17	9
B	15	4
AB	5	1
Duration of HD therapy (months)	-	43.2 ± 37.3; 52; 5–64
Time since KTx (months)	68.9 ± 70.0; 47, 20–102	-
Living/deceased donor	5/49	-
1st/2nd/3rd transplant	46/7/1	-
Current IS		
Steroids	53	0
Tacrolimus/Cyclosporine A	49/5	1/0
MPA/Azathioprine/mTOR/none	39/1/4/10	0/0/0/0
Induction of IS		
Anti-IL-2R	17	-
Anti-CD3	2	-
Comorbidities		
Cardiovascular disease or atrial fibrillation or heart failure	13	17
Hypertension	49	25
diabetes mellitus	13	8
Lung disease	0	1
Chronic liver disease	13	9
Malignancy in history	3	5
Graft function		
Serum creatinine (mg/dL)	1.42 ± 0.47; 1.3, 1.2–1.6	-
eGFR (mL/min/1.73 m^2^, MDRD)	53.5 ± 15.1; 55, 44–59	-
Other medications		
ACEI/ARB	4	8
Statins	17	7
vitamin D	10	22
Smoking	5	0

Data are presented as the number of cases or mean ± SD. KTR: kidney transplant recipients; HD: hemodialysis.

**Table 2:** Anti-SARS-CoV-2 antibody levels in relation to the history of rRT-PCR-confirmed (*n* = 12) or unconfirmed (*n* = 13) SARS-CoV-2 infection in kidney transplant patients.

	rRT-PCR-Confirmed	Unconfirmed	*p*-Value
Number of IgG positive samples	11	13	
IgG (BAU/mL)	821.1 ± 1113.9 369, 207–746	256.5 ± 409.5 111, 64–207	0.016
Number of IgA positive samples	11	12	
IgA (ratio to the cutoff)	28.91 ± 43.34 12.5, 6.3–33.5	13.47 ± 16.58 6.4, 3.4–15.9	0.247
Number of IgM positive samples	0	3	
IgM (ratio to the cutoff)		1.51; 1.92; 3.06	–

Descriptive statistics (mean ± SD, median, and IQR) were calculated for samples classified as positive; when a low number of positive cases were observed, specific values are presented.

**Table 3:** Anti-SARS-CoV-2 antibody levels in relation to the history of rRT-PCR-confirmed (*n* = 11) or unconfirmed (*n* = 3) SARS-CoV-2 infection in hemodialysis patients.

	rRT-PCR-Confirmed	Unconfirmed
Number of IgG positive samples	10	3
IgG (BAU/mL)	551.3 ± 483.8 529, 51–967	106; 534; 635
Number of IgA positive samples	10	3
IgA (ratio to the cutoff)	28.64 ± 46.25 5.4, 3.7–44.3	2.6; 6.2; 19.9
Number of IgM positive samples	1	1
IgM (ratio to the cutoff)	11.5	10.4

Descriptive statistics (mean ± SD, median, and IQR) were calculated for samples classified as positive; when a low number of positive cases were observed, specific values are presented.

**Table 4:** Anti-SARS-CoV-2 cellular response in relation to the history of rRT-PCR-confirmed or unconfirmed SARS-CoV-2 infection in convalescent hemodialysis and kidney transplant patients.

Patients Group	IGRA Response	rRT-PCR-Confirmed	rRT-PCR-Unconfirmed	*p*-Value
HD	mIU/mL *	975.0 ± 736.7 1008, 64–1700	1320; 1700; 1700	0.291
	>64 mIU/mL % (positive/total cases)	72.7% (8/11)	100% (3/3)	
KTR	mIU/mL	617.1 ± 744.7 241, 61–1520	222.1 ± 457.4 53, 23–236	0.207
	>64 mIU/mL % (positive/total cases)	75.0% (9/12)	46.2% (6/13)	

* Descriptive statistics (mean ± SD, median, and IQR); when a low number of cases was analyzed, specific values are presented.

## Data Availability

Detailed data regarding the study would be available from the authors upon request.

## Supplementary Materials

The following are available online at https://www.mdpi.com/article/10.3390/jcm10214833/s1, Table S1: Standard laboratory parameters and inflammatory markers in hemodialysis (HD) and transplant (KTR) patients. Table S2: Standard laboratory parameters and inflammatory markers in hemodialysis (HD) and transplant (KTR) patients in relation to SARS-CoV-2 exposure. Table S3: Phenotypes of peripheral blood T lymphocytes in hemodialysis (HD) and transplant (KTR) patients. Table S4: Phenotypes of peripheral blood B lymphocytes and NK cells in hemodialysis (HD) and transplant (KTR) patients. Table S5: Phenotypes of peripheral blood T lymphocytes in hemodialysis (HD) and transplant (KTR) patients in relation to SARS-CoV-2 exposure. Table S6: Phenotypes of peripheral blood B lymphocytes and NK cells in hemodialysis (HD) and transplant (KTR) patients in relation to SARS-CoV-2 exposure.
