# Supplementary material for: Comparing Humoral and Cellular Adaptive Immunity during Convalescent Phase of COVID-19 in Hemodialysis Patients and Kidney Transplant Recipients
Source: J Clin Med. 2021 Oct 21;10(21):4833. doi: 10.3390/jcm10214833 (PMC8585082; doi:10.3390/jcm10214833)
Supplement: Supplementary file 1 [file jcm-10-04833-s001.zip › jcm-1390672-supplementary.pdf]

Journal of
Clinical Medicine

MDPI

Table S1. Standard laboratory parameters and inflammatory markers in hemodialysis (HD) and transplant (KTR) patients.

Parameter	HD	KTR	*p*-Value
NT-pro-BNP (ng/L)	27866.1 ± 28524.5<br>14975, 3120–70000	447.4 ± 937. 1<br>315, 133–475	< 0.001
BNP (ng/L)	1716.7 ± 3006.5<br>316, 112–2573	78.2 ± 118.6<br>61, 29–98	< 0.001
Albumin (g/dL)	3.60 ± 0.63<br>3.8, 3.3–4.0	4.52 ± 1.59<br>4.3, 4.1–4.6	< 0.001
CRP (mg/L)	17.17 ± 32.90<br>4.4, 2.6–13.7	2.91 ± 5.02<br>1.3, 0.6–3.2	< 0.001
Platelets (10^3/μL)	178.2 ± 60.4<br>184, 127–217	225.7 ± 55.4<br>229, 189–267	0.001
Leucocytes (10^3/μL)	7.15 ± 1.60<br>7.2, 5.8–8.2	8.20 ± 1.98<br>8.1, 6.6–9.7	0.025
Neutrophils (10^3/μL)	5.95 ± 6.25<br>4.7, 3.8–5.6	4.45 ± 1.44<br>4.4, 3.7–5.4	0.510
Lymphocytes (10^3/μL)	1.35 ± 0.52<br>1.4, 1.0–1.5	2.62 ± 0.76<br>2.7, 2.0–3.1	< 0.001
Monocytes (10^3/μL)	0.62 ± 0.20<br>0.7, 0.5–0.7	0.79 ± 0.21<br>0.8, 0.7–1.0	0.002
NLR	4.67 ± 3.53<br>3.9, 2.2–5.9	1.85 ± 0.65<br>1.7, 1.3–2.2	< 0.001
dNLR	1.092 ± 1.451<br>0.84, 0.79–0.87	0.815 ± 0.052<br>0.82, 0.79–0.84	0.267
PLR	149.1 ± 71.7<br>128, 102–186	91.7 ± 30.3<br>88, 73–104	< 0.001
MLR	0.505 ± 0.201<br>0.49, 0.38–0.61	0.322 ± 0.123<br>0.31, 0.25–0.39	< 0.001

Descriptive statistics: mean ± SD, median, IQR

Table S2. Standard laboratory parameters and inflammatory markers in hemodialysis (HD) and transplant (KTR) patients in relation to SARS-CoV-2 exposure.

Parameter	HD			KTR		
	**Convalescent**	**Reference**	***p***	**Convalescent**	**Reference**	***p***
NT-pro-BNP (ng/L)	38885.3 ± 31451.6<br>40662, 7865–70000	16847 ± 21267.8<br>11671, 1603–24264	0.104	589.2 ± 1358.2<br>305, 136–550	325.1 ± 218.1<br>325, 133–455	0.917
BNP (ng/L)	2740.6 ± 3885.1<br>738, 180–3906	599.6 ± 807.7<br>258, 64–730	0.310	96.4 ± 169.6<br>51, 29–100	62.5 ± 37.7<br>69, 23–98	0.931
Albumin (g/dL)	3.33 ± 0.7<br>3.4, 3.1–3.9	3.87 ± 0.42<br>4.0, 3.7–4.2	0.013	4.32 ± 0.31<br>4.3, 4.2–4.6	4.69 ± 2.16<br>4.4, 4.1–4.6	0.937
CRP (mg/L)	28.71 ± 42.99<br>6.5, 3.6–17.7	4.67 ± 4.13<br>3.3, 1.9–4.6	0.041	3.25 ± 6.48<br>1.2, 0.5–2.0	2.61 ± 3.4<br>2.0, 0.6–3.2	0.477
Platelets (10^3/µL)	195.6 ± 69.2<br>193, 127–222	159.3 ± 44.7<br>143, 131–194	0.242	231.5 ± 60.3<br>231, 189–269	220.8 ± 51.4<br>217, 190–239	0.390
Leucocytes (10^3/µL)	7.27 ± 1.83<br>6.9, 5.8–8.5	7.01 ± 1.37<br>7.3, 6.0–8.0	0.807	8.12 ± 2.09<br>8.1, 6.7–9.5	8.27 ± 1.91<br>8.1, 6.6–9.9	0.924
Neutrophils (10^3/µL)	4.82 ± 1.93<br>4.2, 3.6–6.4	7.18 ± 8.84<br>4.9, 3.9–5.6	0.786	4.45 ± 1.43<br>4.4, 3.2–5.2	4.7 ± 1.46<br>4.2, 3.8–5.6	0.869
Lymphocytes (10^3/µL)	1.191 ± 0.484<br>1.12, 0.93–1.42	1.513 ± 0.522<br>1.48, 1.21–1.67	0.109	2.687 ± 0.817<br>2.66, 2.53–3.09	2.568 ± 0.721<br>2.47, 1.95–3.07	0.538
Monocytes (10^3/µL)	0.652 ± 0.196<br>0.69, 0.56–0.74	0.592 ± 0.215<br>0.58, 0.44–0.72	0.355	0.788 ± 0.259<br>0.83, 0.70–0.97	0.784 ± 0.162<br>0.76, 0.66–0.88	0.493
NLR	4.528 ± 2.053<br>4.16, 2.22–5.89	4.823 ± 4.756<br>2.94, 2.33–5.83	0.463	1.749 ± 0.607<br>1.69, 1.25–2.10	1.933 ± 0.684<br>1.89, 1.38–2.31	0.286
dNLR	0.782 ± 0.151<br>0.82, 0.79–0.86	1.429 ± 2.080<br>0.86, 0.80–0.90	0.313	0.814 ± 0.06<br>0.81, 0.79–0.84	0.816 ± 0.046<br>0.82, 0.80–0.84	0.577
PLR	181 ± 75.2<br>156, 128–208	114.5 ± 50.3<br>104, 89–126	0.010	92.4 ± 31.5<br>88, 73–108	91.1 ± 29.8<br>85, 73–100	0.748
MLR	0.592 ± 0.206<br>0.50, 0.49–0.63	0.412 ± 0.154<br>0.39, 0.28–0.52	0.012	0.32 ± 0.154<br>0.28, 0.26–0.35	0.323 ± 0.092<br>0.32, 0.25–0.39	0.526

Descriptive statistics: mean ± SD, median, IQR

Table S3. Phenotypes of peripheral blood T lymphocytes in hemodialysis (HD) and transplant (KTR) patients.

Cell population	HD	KTR	*p*
T cells/μL	1071.3 ± 640.7<br>1003, 726–1116	2112.0 ± 697.3<br>2213, 1565–2643	< 0.001
T %lymphocytes	77.17 ± 8.05<br>79.2, 69.2–82.6	79.63 ± 7.81<br>80.3, 76.0–86.0	0.178
CD4+ T cells /μL	627.8 ± 292.9<br>586, 461–771	1263.9 ± 528.5<br>1186, 811–1725	< 0.001
CD8+ T cells /μL	375.4 ± 302.3<br>272, 172–420	738.6 ± 328.3<br>721, 499–908	< 0.001
CD4+CD8+ T cells /μL	16.10 ± 14.99<br>9.6, 7.2–18.4	47.51 ± 125.10<br>20.4, 9.3–38.1	0.018
CD4+ / CD8+	2.32 ± 1.64<br>1.9, 1.3–2.8	2.11 ± 1.56<br>1.8, 1.2–2.4	0.651
CD4+ %T	60.76 ± 13.76<br>60.1, 55.2–70.8	59.16 ± 12.24<br>60.5, 51.6–67.6	0.702
CD8+ %T	34.00 ± 12.98<br>33.9, 24.8–40.4	35.64 ± 12.49<br>34.0, 28.0–43.3	0.664
CD4+CD8+ %T	1.554 ± 1.355<br>1.06, 0.77–1.72	2.010 ± 4.604<br>0.91, 0.47–1.98	0.233
Regulatory T cells/μL	15.65 ± 10.23<br>13.8, 8.7–20.3	17.78 ± 10.30<br>15.7, 10.1–23.7	0.285
Th1 cells/μL	113.3 ± 65.5<br>108, 69–135	161.8 ± 108.0<br>141, 97–196	0.057
Th2 cells/μL	3.76 ± 2.74<br>3.5, 1.6–4.8	2.48 ± 1.48<br>2.2, 1.3–3.3	0.034
Th17 cells/μL	6.33 ± 4.88<br>4.1, 2.9–10.9	5.84 ± 3.04<br>5.3, 3.6–7.6	0.677
Regulatory T %CD4+ T	2.476 ± 0.828<br>2.39, 1.75–3.05	1.469 ± 0.714<br>1.46, 0.91–1.87	<0.001
Th1 % CD4+ T	18.92 ± 7.80<br>18.3, 12.6–22.3	13.66 ± 8.09<br>11.7, 8.1–17.9	<0.003
Th2 % CD4+ T	0.612 ± 0.400<br>0.47, 0.41–0.72	0.211 ± 0.124<br>0.18, 0.13–0.25	<0.001
Th17 % CD4+ T	1.016 ± 0.578<br>0.89, 0.56–1.34	0.506 ± 0.298<br>0.43, 0.28–0.60	<0.001

Descriptive statistics: mean ± SD, median, IQR

Table S4. Phenotypes of peripheral blood B lymphocytes and NK cells in hemodialysis (HD) and transplant (KTR) patients.

Cell Population	HD	KTR	*p*
B cells/μL	120.4 ± 92.7<br>98, 62–147	200.0 ± 172.6<br>131, 95–287	0.021
B %lymphocytes	9.12 ± 5.66<br>9.5, 4.4–11.9	7.35 ± 4.82<br>6, 3.6–9	0.150
Plasmablasts cells/μL	2.275 ± 2.795<br>0.97, 0.61–2.79	0.809 ± 1.172<br>0.36, 0.12–1.19	< 0.001
IgD- memory B cells/μL	16.34 ± 13.14<br>10.4, 8.3–23.4	48.85 ± 71.87<br>27.6, 15.1–51.5	< 0.001
IgD+ memory B cells/μL	13.14 ± 30.91<br>4.5, 3.0–8.5	30.69 ± 34.30<br>17.2, 9.0–45.6	< 0.001
Naïve B cells/μL	80.5 ± 69.4<br>65, 29–114	110.5 ± 104.7<br>72, 49–142	0.242
Transitional B cells/μL	3.489 ± 4.743<br>1.63, 0.49–4.31	1.489 ± 1.710<br>0.76, 0.33–2.14	0.122
Plasmablasts %B	3.663 ± 5.429<br>1.35, 0.56–2.93	0.594 ± 0.845<br>0.23, 0.08–0.81	<0.001
IgD- memory B %B	16.43 ± 10.16<br>15.1, 8.7–23.2	23.37 ± 15.03<br>19.4, 12.7–31.1	0.042
IgD+ memory B %B	9.46 ± 9.00<br>5.8, 2.9–15.5	15.2 ± 10.29<br>12.5, 6.5–23.2	0.004
Naïve B %B	62.21 ± 19.94<br>67.9, 48.1–78.1	56.04 ± 21.07<br>56.2, 39.1–73.4	0.242
Transitional B %B	2.825 ± 3.796<br>1.53, 0.60–3.71	0.914 ± 1.116<br>0.65, 0.24–0.98	0.002
NK cells/μL	156.1 ± 84.3<br>147, 103–175	321.5 ± 224.9<br>256, 148–448	< 0.001
NK %lymphocytes	13.14 ± 7.89<br>10.5, 8.0–16.7	12.5 ± 8.12<br>10.4, 7.0–17.4	0.592
NKT cells/μL	70.0 ± 87.9<br>42, 23–84	176.8 ± 177.9<br>121, 65–202	< 0.001
NKT %lymphocytes	5.016 ± 3.814<br>3.38, 2.66–7.14	6.381 ± 5.483<br>4.39, 2.31–7.38	0.484

Descriptive statistics: mean ± SD, median, IQR

Table S5. Phenotypes of peripheral blood T lymphocytes in hemodialysis (HD) and transplant (KTR) patients in relation to SARS-CoV-2 exposure.

Cell Population	HD			KTR		
	Convalescent	Unexposed	*p*	Convalescent	Unexposed	*p*
T cells/μL	878.4 ± 412.2 884, 538–1064	1296.5 ± 793.5 1069, 928–1329	0.076	2190.6 ± 684 2283, 1721–2635	2044.3 ± 713.5 1696, 1438–2643	0.476
T %lymphocytes	77.26 ± 7.11 79.2. 74.1–82.6	77.07 ± 9.36 79.0, 69.1–83.2	0.979	80.71 ± 7.67 80.8, 77.9–86.9	78.7 ± 7.94 77.7, 73.7–85.4	0.176
CD4+ T cells /μL	529.0 ± 247.5 583, 342–617	743.1 ± 309.3 764, 539–884	0.105	1308.5 ± 569.8 1343, 803–1711	1225.5 ± 497.1 1050, 824–1725	0.822
CD4+ %T	60.92 ± 15.02 60.1, 55.2–75.7	60.57 ± 12.78 60.7, 54.1–70	0.898	58.75 ± 13.16 59.0, 45.9–68.4	59.51 ± 11.61 61.3, 56.4–67.3	0.788
CD8+ T cells /μL	314.1 ± 236.4 179, 164–400	446.9 ± 362.4 365, 254–458	0.129	758.1 ± 351.6 722, 590–931	721.7 ± 312.1 720, 497–899	0.579
CD8+ %T	34.82 ± 14.7 35.1, 21.7–42.4	33.03 ± 11.2 33.9, 25.7–40.0	0.898	35.58 ± 14.17 32.7, 27.4–49.7	35.69 ± 11.11 34.3, 29.5–38.4	0.896
CD4+CD8+ T cells /μL	8.26 ± 4.24 7.4, 6.5–9.6	25.24 ± 17.84 19.2, 9.9–35.8	<0.001	69.26 ± 180.52 21.5, 10.2–40.6	28.76 ± 30.1 20.1, 8.8–33.7	0.567
CD4+CD8+ %T	0.99 ± 0.40 0.9, 0.7–1.1	2.22 ± 1.76 1.6, 1.0–2.7	0.022	2.77 ± 6.63 0.9, 0.5–1.7	1.35 ± 1.2 0.9, 0.5–2.0	0.671
CD4+ / CD8+	2.35 ± 1.68 1.8, 1.3–3.5	2.28 ± 1.66 1.9, 1.3–2.7	0.979	2.33 ± 2.06 1.8, 0.9–2.5	1.92 ± 0.93 1.8, 1.5–2.3	0.979
CD4+ CD28- CD57- T cells /μL	12.44 ± 15.74 7.8, 1.2–16.6	5.47 ± 4.86 3.7, 2–8.4	0.527	12.04 ± 16.57 4.3, 0.5–17.7	12.48 ± 28.04 3.5, 0.8–10.9	0.767
CD4+ CD28- CD57- %T	3.56 ± 6.43 1.6, 0.2–3.3	0.93 ± 1.08 0.5, 0.3–1.1	0.231	0.96 ± 1.39 0.4, 0.1–1.2	1.1 ± 2.22 0.3, 0.1–1	0.905
CD8+ CD28- CD57- T cells /μL	19.13 ± 20.7 15.4, 3.5–28.4	36.13 ± 33.75 33.3, 5–58.9	0.176	62.65 ± 108.59 25.5, 4.8–79.2	45.28 ± 66.88 21.1, 2.4–48.7	0.618
CD8+ CD28- CD57- %T	5.02 ± 5.27 3.9, 0.6–6.5	4.83 ± 3.77 5.4, 0.9–7.6	0.781	4.92 ± 6.95 2.1, 0.4–6.5	4.24 ± 6.86 1.4, 0.3–6.6	0.606
CD4+ CD28- CD57+ T cells /μL	34.25 ± 35.02 23.3, 12.8–51.2	70.43 ± 84.17 32.1, 13.2–105.8	0.347	156.67 ± 164.68 95.0, 33.8–223.8	106.53 ± 113.14 76.5, 31.8–137.1	0.309
CD4+ CD28- CD57+ %T	11.26 ± 7.97 8.4, 4.9–14.7	14.79 ± 11.61 11.6, 7.7–20.7	0.560	22.66 ± 35.31 15.2, 5.5–22.6	12.84 ± 8.39 10.7, 6.6–16.6	0.247
CD8+ CD28- CD57+ T cells /μL	138.41 ± 161.63 61.9, 39.9–128	135.17 ± 177.43 60.6, 26.1–160.7	0.781	239.26 ± 166.57 196.9, 107.8–372.9	236.68 ± 176.45 169.0, 115.3–369.7	0.877
CD8+ CD28- CD57+ %T	37.15 ± 21.71 33.6, 15.1–56.8	25.65 ± 17.97 22.7, 15.4–36.6	0.231	33.33 ± 25.95 28.7, 19.1–37.8	30.13 ± 12.86 29.8, 20.4–38.8	0.918
Regulatory T cells/μL	12.75 ± 6.45 13.8, 7.6–16.7	19.03 ± 12.86 15.4, 9.6–24.2	0.227	19.42 ± 12.18 15.0, 11.7–25.8	16.36 ± 8.32 16.3, 9.8–19.9	0.488
Regulatory T %CD4+ T	2.452 ± 0.688 2.39, 1.97–2.86	2.504 ± 0.999 2.30, 1.63–3.55	0.898	1.568 ± 0.833 1.49, 0.91–1.85	1.383 ± 0.595 1.34, 0.91–1.87	0.566
Th1 cells/μL	90.5 ± 45.6 76, 57–122	140 ± 76.6 126, 98–166	0.076	171 ± 114.8 142, 99–204	154.0 ± 103.1 140, 97–176	0.715
Th1 % CD4+ T	18.92 ± 9.14 17.5, 12.4–30.3	18.91 ± 6.28 18.3, 14.6–21.5	0.857	14.07 ± 7.61 14.3, 7.6–17.9	13.3 ± 8.59 11.0, 8.3–15.5	0.633
Th2 cells/μL	3.79 ± 3.27 3.5, 1.4–4.8	3.72 ± 2.10 3.6, 2.2–4.8	0.797	2.57 ± 1.49 2.2, 1.7–3.1	2.4 ± 1.5 2.1, 1.2–3.3	0.555
Th2 % CD4+ T	0.72 ± 0.509 0.58, 0.4–0.97	0.486 ± 0.158 0.47, 0.43–0.6	0.471	0.216 ± 0.113 0.19, 0.14–0.26	0.207 ± 0.135 0.17, 0.12–0.24	0.573
Th17 cells/μL	4.67 ± 3.35 3.8, 2.8–5.2	8.27 ± 5.76 5.4, 3.6–12.8	0.080	5.49 ± 2.52 5.3, 3.5–7.2	6.14 ± 3.44 5.4, 3.8–8.3	0.450
Th17 % CD4+ T	0.922 ± 0.526 0.82, 0.56–1.18	1.126 ± 0.639 1.26, 0.53–1.55	0.456	0.488 ± 0.307 0.41, 0.28–0.59	0.522 ± 0.295 0.49, 0.36–0.60	0.646

Descriptive statistics: mean ± SD, median, IQR

Table S6. Phenotypes of peripheral blood B lymphocytes and NK cells in hemodialysis (HD) and transplant (KTR) patients in relation to SARS-CoV-2 exposure.

Cell Population	HD			KTR		
	**Convalescent**	**Unexposed**	***p***	**Convalescent**	**Unexposed**	***p***
B cells/μL	103.2 ± 94.8<br>85, 62–108	140.4 ± 89.9<br>135, 68–203	0.172	215.0 ± 191.6<br>166, 101–286	187.0 ± 156.6<br>124, 86–287	0.499
B %lymphocytes	8.89 ± 5.63<br>7.7, 5.6–10.4	9.39 ± 5.93<br>11.2, 3.6–12.5	0.487	7.41 ± 4.13<br>7.3, 3.8–9.0	7.30 ± 5.41<br>5.0, 3.6–9.0	0.445
Plasmablasts cells/μL	2.804 ± 3.336<br>1.34, 0.73–3.78	1.658 ± 1.956<br>0.92, 0.52–1.62	0.396	0.882 ± 1.243<br>0.42, 0.14–0.81	0.746 ± 1.126<br>0.22, 0.12–1.19	0.526
Plasmablasts %B	4.32 ± 4.84<br>1.6, 0.6–9.4	2.9 ± 6.17<br>1.0, 0.4–2.2	0.247	0.61 ± 0.82<br>0.3, 0.1–0.8	0.58 ± 0.88<br>0.2, 0.1–0.7	0.952
IgD- memory B cells/μL	11.9 ± 8.25<br>8.9, 7.2–16.8	21.5 ± 16.05<br>15.1, 8.9–33.3	0.105	56.74 ± 91.82<br>30.5, 15.1–57.0	42.05 ± 49.46<br>25.8, 16.3–42.3	0.567
IgD- memory B %B	14.18 ± 8.31<br>10.8, 7.9–18.3	19.06 ± 11.79<br>15.9, 11.0–25.0	0.292	22.76 ± 12.39<br>18.9, 14.4–30.2	23.88 ± 17.20<br>19.8, 10.6–31.5	0.822
IgD+ memory B cells/μL	16.69 ± 41.17<br>4.6, 2.5–8.5	9.00 ± 11.56<br>4.3, 3.1–8.1	0.857	33.88 ± 37.06<br>19.8, 7.4–58.9	27.94 ± 32.15<br>15.0, 9.5–33.7	0.652
IgD+ memory B %B	10.64 ± 10.69<br>5.8, 4.1–15.5	8.09 ± 6.71<br>4.9, 2.3–14.5	0.425	15.51 ± 10.35<br>13.6, 6.1–24.5	14.94 ± 10.42<br>10.6, 7.5–20.5	0.979
Naïve B cells/μL	62.76 ± 52.19<br>53, 28.6–76	101.3 ± 82.82<br>96.2, 31.9–138	0.172	113.12 ± 97.42<br>79.1, 50.6–152.0	108.29 ± 112.26<br>65.8, 44.1–87.8	0.415
Naïve B %B	61.39 ± 18.73<br>59.8, 49.2–80.7	63.18 ± 22.07<br>69.1, 47.0–76.9	0.857	55.97 ± 21.23<br>56.0, 41.0–70.1	56.11 ± 21.31<br>56.4, 39.1–74.2	0.890
Transitional B cells/μL	2.923 ± 4.451<br>1.63, 0.49–2.76	4.143 ± 5.181<br>2.13, 0.45–6.47	0.738	1.754 ± 1.658<br>1.10, 0.44–2.79	1.261 ± 1.749<br>0.55, 0.32–1.23	0.176
Transitional B %B	3.112 ± 4.497<br>1.70, 1.20–3.32	2.489 ± 2.935<br>1.03, 0.58–4.21	0.520	1.104 ± 1.317<br>0.70, 0.58–1.01	0.750 ± 0.902<br>0.43, 0.24–0.87	0.274
NK cells/μL	133.5 ± 63.2<br>142, 82–162	182.4 ± 100.2<br>165, 114–185	0.269	310.9 ± 265.9<br>204, 108–434	330.7 ± 187.0<br>313, 169–448	0.367
NK %lymphocytes	13.23 ± 7.05<br>12.4, 8.0–16.7	13.03 ± 9.1<br>9.9, 8.0–15.1	0.777	11.41 ± 8.25<br>10.4, 3.2–15.2	13.44 ± 8.04<br>10.5, 7.1–18.5	0.362
NKT cells/μL	50.8 ± 35.5<br>36, 23–78	92.4 ± 122.7<br>49, 23–109	0.589	158.4 ± 162.9<br>117, 58–185	192.7 ± 191.2<br>124, 69–202	0.521
NKT %lymphocytes	5.06 ± 3.74<br>3.2, 2.7–7.0	4.97 ± 4.06<br>3.4, 2.0–7.9	0.817	5.69 ± 5.46<br>4.0, 2.1–7.3	6.98 ± 5.53<br>5.4, 2.6–7.4	0.252

Descriptive statistics: mean ± SD, median, IQR
